# Necrology

**Published:** 1896-08

**Authors:** 


### NECROLOGY.

### PROFESSOR JAMES HAMILL.

The veterinary profession and the horse have lost one of their best and greatest friends in the death of this noble and true man.

Dr. Hamill died of cerebral apoplexy in court on May 29, 1896, while acting as an expert witness in a horse case. He was called to the stand to be examined, and was partly through with his testimony when he suddenly fell from his chair and expired in a few minutes.

Dr. Hamill was fifty-five years old, was born in Bellicastle, County Antrim, Ireland, of Scotch parents, and came to this country while a very young man. He was a born horseman, a trait which he inherited from his father, who was also a skilled horseman and an experienced shoeingsmith. He graduated from the Columbia and the New York Colleges of Veterinary Surgeons, and taught for many years in the above colleges. His lectures were spontaneous—never used notes; had a splendid flexible voice; was an orator, but never boisterous. Every student that had the good fortune to hear him benefited by his beautiful delivery and truthful advice. He was an expert on the horse's foot, an excellent practitioner, and a very good surgeon. His knowledge of the anatomy, physiology, and pathology of the horse's foot was marvellous; he was an advanced thinker and highly educated. If his good deeds were placed in print, this JOURNAL would not hold them. There was no limit to his good-will to his fellow-man; he was faithful, kind, and sympathetic; his left hand never knew what his right hand did; he perhaps did as much to advance and improve the veterinary profession as any man; he was always ready to assist a friend; it was no trouble for him to go one mile or a hundred if he could only do some good deed. There are thousands who bear witness to this. His friends were numerous; his enemies few, if any.

Dr. Hamill is mourned by a host of professional and non-professional friends throughout this country and Europe. His knowledge and honest endeavor can be seen in Manger's beautiful work on the horse, of which a large portion came from his pen, and in the new standard dictionary everything pertaining to the horse also came from his pen. Dr. Hamill seemed too modest to write a book, so his great skill and extensive knowledge of the horse and his foot is therefore lost.

Dr. Hamill was an expert shoeingsmith. Years ago his practice became so large that he gave up shoeing and devoted his whole attention to the practice of veterinary medicine. Dr. Hamill was an equine specialist, pure and simple; he would not treat any other animal. There was no reason for him not becoming a general practitioner except that he argued that each and every medical man should be a specialist, as no man could do himself justice in general practice.

Dr. Hamill's religion was a power in itself; he was humiliated to all mankind and a martyr to his friends. I am safe in saying that with his vast knowledge this great and peculiar character was rarely understood.

He leaves a widow and five children to mourn his loss. The interment took place in the family plot at Greenwood, among his loved ones and friends. J. H. Gardner, D.V.S.

### David Roberge.

David Roberge died on Thursday, July 9th, at his late residence, Van Sicklen Station, Coney Island, N. Y., in the seventy-second year of his age. He was born in Montreal, Canada, his father being a conservative farmer. He was married, in 1850, to Miss Sarah E. Stowe, a lady of much culture and education. They reared a family of eleven children, nine of whom are still living. During his youth and early manhood he took great delight in the study of the horse. His mind being of a mechanical turn, he conceived the idea of treating lame horses mechanically instead of medicinally, and carried this idea to such a degree of perfection that he was not only in the first line, but vastly in advance of all men, in his chosen profession. Retiring in disposition, he cultivated the friendship of few, but among them may be mentioned Robert Bonner, whose friendship for him increased yearly, as was shown by their constant association for some twenty-seven years in the study of the horse's foot. Fertile in ideas, strong in argument, and persistent in effort, he overcame every obstacle in his way, and at last gained the top of the ladder in his line. He learned the English language by the aid of his wife after they came to New York, although in conversation he always retained the accent learned in the French college from which he was graduated. He was the author of *The Foot of the Horse*, a book dedicated to Robert Bonner, in which he crowded the results of his experiments during his life, and which will go down to posterity as the greatest benefactor of the equine race. Mr. Roberge was very fond of road-driving, and could daily be seen on the road in the times of Commodore Vanderbilt, William H. Vanderbilt, Capt. Jacob Vanderbilt, Messrs. Turnbull, Forster, Dewey, and others who took so much pleasure riding behind their famous roadsters. His skill at driving was second only to his skill in shoeing horses. Two years ago he was called to the Chair of Foot Pathology in the New York College of Veterinary Surgeons, and only relinquished his labors at teaching on account of ill-health. His many devices for correcting an unbalanced foot—to which cause he attributed all lameness common to the horse—were patented in this and other countries, and entirely abolished the use of blisters, firing-irons, and setons in the treatment of lame horses. His death is mourned by all true lovers of the horse, who unitedly and sadly bow to bedeck his memory with a wreath of forget-me-nots. At his funeral Robert Bonner, Mr. Busby, of the *Turf, Field, and Farm*, Mr. Farmer, and Dr. J. H. Ferster were honorary pall-bearers.

J. H. Ferster, V. S.

The many friends of Dr. Thomas B. Rayner, of Chestnut Hill, Philadelphia, will deeply sympathize with him in the loss of his younger son, Moncure R. Rayner, from acute tuberculosis on the morning of July 6th. This son, the promised successor of his father, whose education was already in progress to fit him for the veterinary profession, had spent one year in the Biological Department and two years in the Veterinary Department of the University of Pennsylvania, where he had attained a high rank as an interested and devoted student, and gave great promise to be a worthy successor of his father. Just attaining the age of manhood, and filled with the energy and ambition of youth, with a great future before him, it seemed doubly hard for him to yield to the ravages of that ever-deceitful malady. It was a sad blow to the parents, and one which every acquaintance of the family will share with them.

Dr. W. E. Smith, of Sedalia, Mo., graduate of the American Veterinary College, Class of '91, died recently of diabetes.

On to Buffalo!

